# ENGAGING DEMENTIA CARE PARTNERS IN FALL RISK MANAGEMENT: HEALTH PROFESSIONALS’ PERSPECTIVES

**DOI:** 10.1093/geroni/igae098.0061

**Published:** 2024-12-31

**Authors:** Yuanjin Zhou, Amanda Kilcrease, Elizabeth Phelan

**Affiliations:** The University of Texas Austin, Austin, Texas, United States; The University of Texas Austin, Austin, Texas, United States; University of Washington, Seattle, Washington, United States

## Abstract

Older people with dementia are at higher risk of falls than those without dementia. Informal care partners are well-positioned to assist them in reducing their fall risk. However, little is known about what support care partners may need to accomplish this. Health professionals with experience working with older people with dementia and their care partners may have important insights. This study thus aimed to characterize barriers for care partners to engage in fall risk management (FRM) from health professionals’ perspectives and identify strategies to overcome these barriers. Using snowball sampling, we conducted semi-structured, qualitative interviews via Zoom with 22 health professionals from six disciplines (geriatric medicine, nursing, occupational therapy, physical therapy, social work, and pharmacy). These health professionals had an average of 22 (range: 5-44) years of clinical experience in dementia care. Thematic analysis identified the following themes related to barriers: care partners’ care burden, a low perceived need for FRM, a tendency to prioritize the preferences of older people, resistance to making changes, disagreement about care arrangement, and challenges accessing needed resources (e.g., food, home safety evaluation, mobility devices). Suggested strategies included addressing care partners’ primary concerns first (e.g., behavioral issues of older people with dementia), assessing care partners’ motivation, existing strategies, and potential support for FRM, linking recommendations to priorities and goals of the dyads, and connecting them with appropriate resources. This study offers new perspectives on challenges dementia care partners may face in managing fall risk. These perspectives warrant further exploration with dementia care partners.

